# Comparative QSPR study of food preservatives using topological indices and regression models

**DOI:** 10.1038/s41598-025-08002-5

**Published:** 2025-07-08

**Authors:** K. B. Gayathri, S. Roy

**Affiliations:** Department of Mathematics, School of Advanced Sciences, Vellore Institute of Technology, Vellore, 632 014 India

**Keywords:** Topological indices, QSPR study, Curvilinear regression models, Food preservatives, Chemical biology, Chemistry, Mathematics and computing

## Abstract

Food preservatives play a crucial role in extending the shelf life of food products. Understanding their physicochemical properties can help in designing more effective and safer preservatives. In this study, we use a Quantitative Structure Property Relationship (QSPR) approach based on topological indices to develop a predictive model for certain physicochemical properties of food preservatives. We compare the performance of linear and curvilinear regression models to understand which provides the best prediction model. Among the tested models, the cubic regression model demonstrated superior predictive performance. Of all the models tested, the cubic regression model had the best predictive capabilities such as $R^2 = 0.9998$ for vapour density and $R^2 = 0.9039$ for molecular weight. To validate our findings, we employ the developed model to estimate the properties of an existing food preservative, the propionic acid. Our results offer valuable insights that can aid in the development of new and improved food preservatives.

## Introduction

Food additives are substances added to food to enhance its shelf life, nutritional value, flavour, or appearance. These substances can be classified as colorants, emulsifiers, flavour enhancers, antioxidants, preservatives, and sweeteners^1,2^. Natural or synthetic substances added to food to prolong its shelf life are known as food preservatives^3,4^. These compounds function as antibacterial agents, lessen oxidation, and inhibit the generation of toxins. Up to 40% of all food produced for human consumption globally is wasted due to microbial food deterioration. Food safety and edible quality are ensured by the addition of preservatives^5^. The demand for less hazardous preservatives is increasing. As a result, the market for safe and effective preservatives is growing. With increasing demand, developing and evaluating new preservatives is imperative^6^. Analyzing the physicochemical properties of the compound is crucial for its effectiveness. Predictive models use computational and mathematical techniques without the aid of extensive laboratory testing setups to estimate the biological effects. Here we develop models which help to assess the toxicity, allergenicity, and metabolic effects using the structural and physicochemical properties^7,8^.

QSPR (Quantitative Structure Property Relationship) and QSAR (Quantitative Structure-Activity Relationship) are computational modeling techniques that use a molecule’s chemical structure to predict its physicochemical properties and biological activity, respectively^9,10^. With the use of certain molecular descriptors and statistical or machine learning models, QSAR establishes a relationship between a compound’s chemical structure and biological activities, such as toxicity or medicinal efficacy. Rather, QSPR focuses on using the structure to predict physical or chemical aspects like melting or boiling points. Both approaches use quantitative analysis of topological properties to generate prediction models that support material science, environmental chemistry, and drug design^11–13^.

The study of networks of points connected by lines is the subject of the mathematical field known as graph theory^14^. The application of graph theory can help solve several real-world problems. Over the nineteenth century, graph theory was used in chemistry. The development of chemical graph theory, a new subfield of graph theory, followed. Chemical graphs are used to depict chemical structures in chemical graph theory^15^. An atom is represented by a vertex, and the relationship between atoms is represented by an edge. The compound’s chemical structure is linked to the majority of its chemical information. A chemical network is represented by topological indices (TI), which are numerical invariants. The TIs are utilized in QSAR and QSPR research to build models that forecast the physical, chemical, or biochemical properties of certain compounds^16–19^. As a computational method for drug design and various other structural analysis, this concept is widely applied^20–26^.

Several studies have used different topological indices to predict specific properties of compounds. In 2002, scientists used certain specific TIs to forecast the biological activity of particular alkoxyphenols^27^. To forecast the physical characteristics of medications for mental disorders, Ejma et al. utilized ensemble learning in conjunction with topological indices^28^. Using regression models and topological indices, Huili Li et al. examined the structural characteristics of amino acids in order to forecast their chemical and physical characteristics^29^. The physical characteristics of some antibacterial medications were recently predicted by Abubakar et al. using two regression models and specific neighborhood sum-based indices^30^.

Both linear regression models and non-linear regression models can be utilized to execute QSAR and QSPR analysis^31,13^. The link between the molecular structural property and the desired activity or property determines the type of model to use. When the relationship between the dependent and independent variables is non-linear, curvilinear models are thought to be optimal. In this study, the physicochemical properties (P) of the food preservatives are the dependent variables, and their corresponding topological indices (TI) are the independent variables. Here we consider linear, quadratic and cubic regression models.

Linear:

$$\begin{aligned} P=a_1(TI)+a_2 \end{aligned}$$

Quadratic:

$$\begin{aligned} P=a_3(TI)^2+a_4(TI)+a_5 \end{aligned}$$

Cubic:

$$\begin{aligned} P=a_6(TI)^3+a_7(TI)^2+a_8(TI)+a_9 \end{aligned}$$

where a_*i*_’s ($i=1$ to 9) are constants.

## Materials and methods

**Fig. 1:**
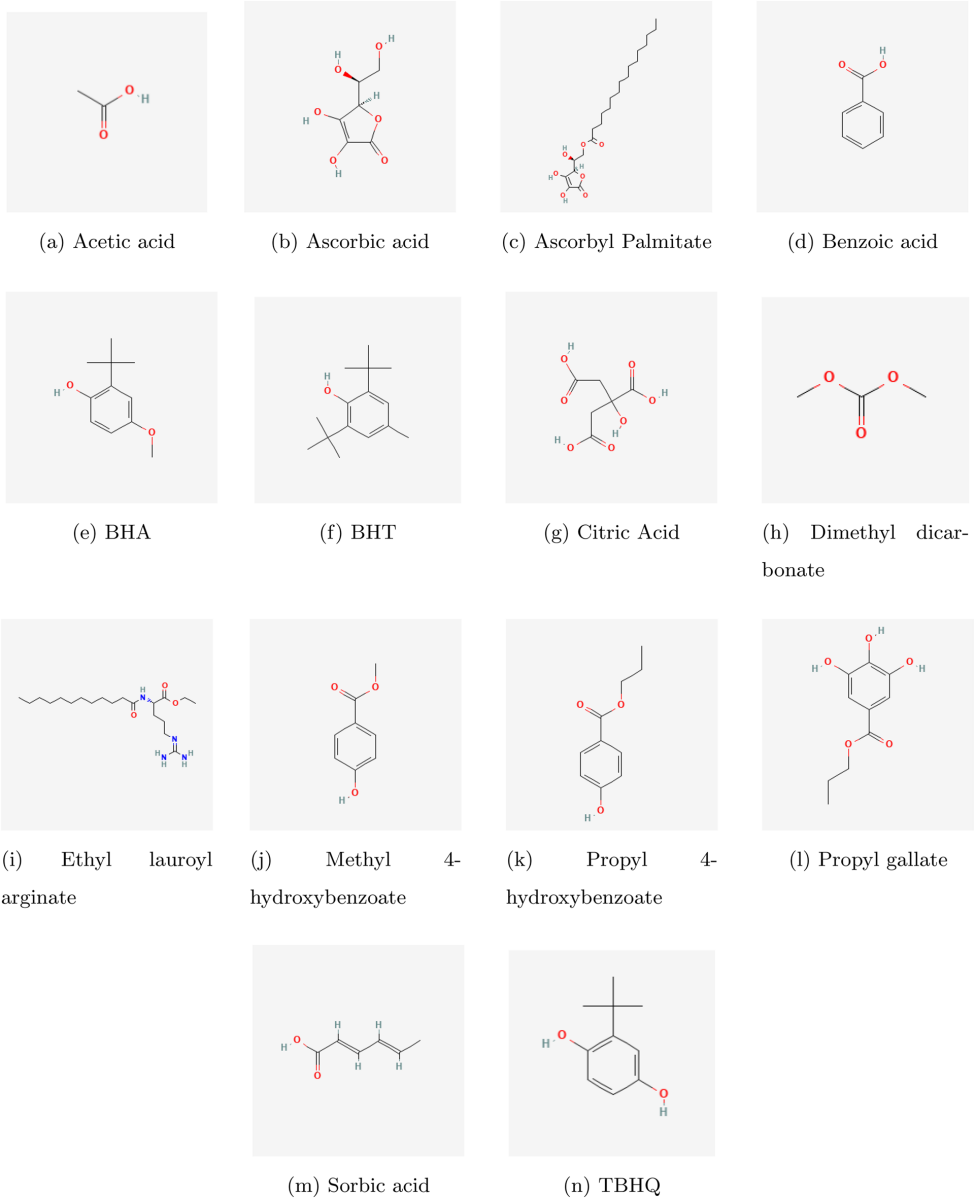
Molecular structures of food preservatives.

The study takes into account fourteen chemical compounds that are currently used for efficient food preservation. Acetic acid is a commonly used preservative as it breaks down bacterial and fungal cell membranes, making it impossible for them to survive. It is also vinegar’s primary ingredient. It is a widely accepted chemical that occurs naturally^32^. Ascorbic acid is a naturally occurring antioxidant that is mostly added to fruits, vegetables, and drinks, in contrast to other preservatives^33^. An ester derivative of ascorbic acid is ascorbyl palmitate. It is added to sunflower oil to extend its shelf life^34^. One of the earliest chemical preservatives still in use in the food enterprises is benzoic acid. It works well to stop the growth of yeast^35^. Generally regarded as antibacterials, butylated hydroxyanisole (BHA) and butylated hydroxytoluene (BHT) are phenolic antioxidants^36^. As an anti-enzymatic preservative, citric acid slows down food from going through enzymatic reactions long after it has been harvested^37^. A chemical preservative called dimethyl carbonate is used to prolong the shelf life of goods like salsa^38^. A cationic surfactant called ethyl lauroyl arinate is used in food preservation to enhance the microbiological safety and quality characteristics of a variety of food products^39^. Often referred to as methylparaben, methyl 4-hydroxybenzoate is a typical food preservative used in baked goods and jams^40^. Likewise, processed meat and dairy products include propyl 4-hydroxybenzoate, also referred to as propylparaben, as a food preservative^41^. A common phenolic antioxidant in the food, cosmetics and pharmaceutical sectors is propyl gallate^42^. Sorbic acids and their salts are the most widely used preservatives in the food industry due to their neutral flavor and physiological inertness^43^. Sunflower oil is also preserved using tertiary butylhydroquinone (TBHQ)^44^. Figure 1 shows the molecular structures of the food preservatives mentioned above.

**Fig. 2:**
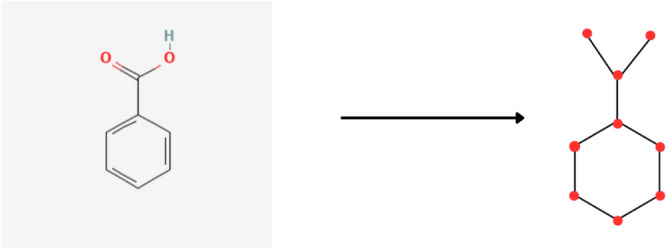
Molecular graph of Benzoic acid.

Figure 2 shows the molecular graph representation of benzoic acid’s chemical structure. Similarly all of the aforementioned compounds’ chemical structures can be depicted as molecular graphs.

The efficacy, stability, and safety of a food preservative compound depend much on several physical and chemical properties, including molecular weight (MW), boiling point (BP), melting point (MP), pKa value, vapour density (VD), log P and LD50 value. A greater LD50 value suggests less toxicity, so the preservative is safer for use. Table 1 depicts the physicochemical properties and LD50 value of the selected food preservatives. All the data are collected from PubChem^45^.

**Table 1:** Physicochemical properties and LD50-value of food preservatives.

Compound	Molecular weight (g/mol)	Melting $\hbox {point}^\circ (\hbox {C})$	Boiling point at 760 mmHg	pKa	Vapour density	log P	LD50 (oral, rat) (mg/kg)
Acetic acid	60.05	16.6	117.9	–	2.07	− 0.17	3310
Ascorbic acid	176.12	191	552.7	4.7	–	− 1.85	11900
Ascorbyl Palmitate	414.5	116.5	–	–	–	–	–
Benzoic acid	122.12	122.4	249.2	4.207	4.21	1.87	1700
BHA	360.494	51	267	–	–	–	–
BHT	220.35	70.5	264.5	12.23	7.6	5.32	2930
Citric acid	192.12	156	310	2.79	–	− 1.64	5500
Dimethyl Dicarbonate	134.09	17	175	–	–	–	260
Ethyl Lauroyl Arginate	384.6	–	–	–	–	–	–
Methyl 4-hydroxybenzoate	152.98	125.2	275	–	–	1.96	5600
Propyl 4-hydroxybenzoate	180.2	97.5	294.5	–	–	3.04	–
Propyl gallate (PG)	212.199	150	–	7.94	7.3	1.8	.
Sorbic acid	112.13	134.5	228	4.76	3.87	1.33	7360
TBHQ	166.217	128	273	10.8	.	–	–

**Fig. 3:**
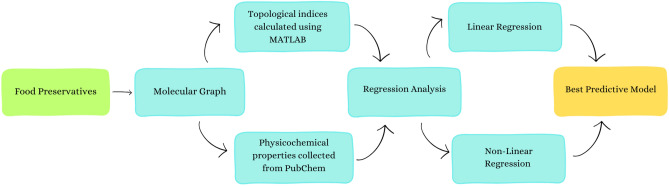
Methodological framework of the study.

The study’s methodology flow chart is presented in Fig. 3.

Let *G* be a simple connected graph with vertex set *V*(*G*) and edge set *E*(*G*). Let *u* be any arbitrary vertex in the set *V*(*G*). Then $d_u$, the degree of the vertex *u*, is the number of vertices adjacent to *u*. To examine the molecular structure of the above mentioned compounds, we employ sixteen degree based indices. Table 2 shows the selected topological indices used for the study.

For calculating the topological indices, we take the aid of the edge partitioning method. This is a useful technique for computing topological indices by categorizing edges based on the degree of their end vertices. Let $E_{(r,s)}$ represent the set of edges joining the vertices *u* and *v*. Here, *r* and *s* represent the degree of vertices *u* and *v*, respectively. For every chemical graph, the maximum degree of a vertex is 4. Here in this study, the molecular graphs of the selected compounds have edges of types $E_{(1,2)},E_{(1,3)},E_{(1,4)},E_{(2,2)},E_{(2,3)},E_{(2,4)},E_{(3,3)},E_{(3,4)}$ . $|E_{(r,s)}|$ represents the cardinality of the set $E_{(r,s)}$. Table 3 gives the edge partitions of the dataset of compounds.

**Table 2:** Topological indices.

Topological index	Notation	Formula	References
First Zagreb Index	$M_1(G)$	$\displaystyle \sum _{uv\in E(G)}(d_u+d_v)$	Gutman and Trinjstic^46^
Second Zagreb Index	$M_2(G)$	$\displaystyle \sum _{uv\in E(G)}(d_ud_v)$	Gutman and Trinjstic^46^
Reduced Zagreb Index	$RM_2(G)$	$\displaystyle \sum _{uv\in E(G)}(d_u-1)(d_v-1)$	Furtula et al.^47^
Hyper Zagreb Index	*HM*(*G*)	$\displaystyle \sum _{uv\in E(G)}(d_u+d_v)^2$	Shirdel et al.^48^
Augmented Zagreb Index	*AZ*(*G*)	$\displaystyle \sum _{uv\in E(G)}\left[ \frac{d_ud_v}{d_u+d_v-2}\right] ^3$	Ali et al.^49^
Randić Zagreb Index	*R*(*G*)	$\displaystyle \sum _{uv\in E(G)}\frac{1}{\sqrt{d_ud_v}}$	Randić^50^
Reciprocal Randić Zagreb Index	*RR*(*G*)	$\displaystyle \sum _{uv\in E(G)}\sqrt{d_ud_v}$	Gutman et al.^51^
Reduced Reciprocal Randić Index	*RRR*(*G*)	$\displaystyle \sum _{uv\in E(G)}\sqrt{(d_u-1)(d_v-1)}$	Gutman et al.^51^
Harmonic Index	*H*(*G*)	$\displaystyle \sum _{uv\in E(G)}\frac{2}{d_u+d_v}$	Fajtlowicz^52^
Sum Connectivity Index	*SC*(*G*)	$\displaystyle \sum _{uv\in E(G)}\frac{1}{\sqrt{d_u+d_v}}$	Trinjastic^53^
Geometric Arithmetic Index	*GA*(*G*)	$\displaystyle \sum _{uv\in E(G)}\frac{2\sqrt{d_ud_v}}{d_u+d_v}$	Vukicevic et al.^54^
Inverse Sum Index	*IS*(*G*)	$\displaystyle \sum _{uv\in E(G)}\frac{d_ud_v}{d_u+d_v}$	Vukicevic et al.^55^
Forgotten Index	*F*(*G*)	$\displaystyle \sum _{uv\in E(G)}d_u^2+d_v^2$	Gutman and Trinjstic^46^
Symmetric Division Index	*SD*(*G*)	$\displaystyle \sum _{uv\in E(G)}\frac{d_u^2+d_v^2}{d_ud_v}$	Vukicevic et al.^55^
Atom Bond Connectivity Index	*ABC*(*G*)	$\displaystyle \sum _{uv\in E(G)}\sqrt{\frac{d_u+d_v-2}{d_ud_v}}$	Estrada et al.^56^
Sombor Index	*So*(*G*)	$\displaystyle \sum _{uv\in E(G)}\sqrt{d_u^2+d_v^2}$	Gutman^57^

**Table 3:** Edge partitions of the compounds.

Compound	$|E_{(1,2)}|$	$|E_{(1,3)}|$	$|E_{(1,4)}|$	$|E_{(2,2)}|$	$|E_{(2,3)}|$	$|E_{(2,4)}|$	$|E_{(3,3)}|$	$|E_{(3,4)}|$
Acetic acid	–	3	–	–	–	–	–	–
Ascorbic acid	1	4	–	–	3	–	4	–
Ascorbyl Palmitate	1	5	–	14	5	–	4	–
Benzoic acid	–	2	–	4	2	–	1	–
BHA	1	1	3	1	5	–	1	1
BHT	–	2	6	–	4	–	2	2
Citric acid	–	6	1	–	2	2	–	1
Dimethyl Dicarbonate	2	2	–	–	4	–	–	–
Ethyl Lauroyl Arginate	2	4	–	13	6	–	1	–
Methyl 4-hydroxybenzoate	1	2	–	2	5	–	1	–
Propyl 4-hydroxybenzoate	1	2	–	4	5	–	1	–
Propyl gallate (PG)	1	4	–	2	5	–	3	–
Sorbic acid	1	2	–	3	1	–	–	–
TBHQ	–	2	3	1	3	–	1	1

## Results and discussion

In this section, we computed the topological indices of all selected compounds. As a representative example, we consider the case of benzoic acid, and the results are summarized in the following theorem.

### **Theorem 3.1**

*Let* ${\mathcal {G}}$ *represent the molecular graph of benzoic acid. Then,* (1) $M_1({\mathcal {G}}) = 40$, (2) $M_2({\mathcal {G}}) = 43$, (3) $RM_2({\mathcal {G}}) = 12$, (4) $HM({\mathcal {G}}) = 182$, (5) $AZ({\mathcal {G}}) = 66.1406$, (6) $R({\mathcal {G}}) = 4.3045$, (7) $RR({\mathcal {G}}) = 19.3631$, (8) $RRR({\mathcal {G}}) = 8.8284$, (9) $H({\mathcal {G}}) = 4.1333$, (10) $SC({\mathcal {G}}) = 4.3027$, (11) $GA({\mathcal {G}}) = 8.6916$, (12) $IS({\mathcal {G}}) = 9.4$, (13) $F({\mathcal {G}}) = 96$, (14) $SDI({\mathcal {G}}) = 21$, (15) $ABC({\mathcal {G}}) = 6.5423$, (16) $So({\mathcal {G}}) = 29.0926$.

### *Proof*

Let ${\mathcal {G}}$ denote the molecular graph of benzoic acid. Then evaluating each indices from Table 2, using the edge partition from Table 3, we get,

- $$M_1({\mathcal {G}}) = 2(1+3) + 4(2+2) + 4(2+3) + 1(3+3) = 40$$
- $$M_2({\mathcal {G}}) = 2(1\times 3) + 4(2\times 2) + 4(2\times 3) + 1(3\times 3) = 43$$
- $$RM_2({\mathcal {G}}) = 2(0\times 2) + 4(1\times 1)+ 4(1\times 2) + 1(2\times 2) = 12$$
- $$HM({\mathcal {G}}) = 2(1+3)^3 + 4(2+2)^3 + 4(2+3)^3 + 1(3+3)^3 = 182$$
- $$AZ({\mathcal {G}}) = 2 \left( \frac{1\times 3}{1+3-2}\right) ^3 + 4 \left( \frac{2\times 2}{2+2-2}\right) ^3 + 4 \left( \frac{2\times 3}{2+3-2}\right) ^3+ 1 \left( \frac{3\times 3}{3+3-2}\right) ^3 = 66.1406$$
- $$R({\mathcal {G}}) = 2 \left( \frac{1}{\sqrt{1\times 3}}\right) + 4 \left( \frac{1}{\sqrt{2\times 2}}\right) + 4 \left( \frac{1}{\sqrt{2\times 3}}\right) + 1 \left( \frac{1}{\sqrt{3\times 3}}\right) = 4.3045$$
- $$RR({\mathcal {G}}) = 2\sqrt{1\times 3} + 4\sqrt{2\times 2}+ 4\sqrt{2\times 3} + 1\sqrt{3\times 3} = 19.3631$$
- $$RRR({\mathcal {G}}) = 2\sqrt{0\times 2}+ 4\sqrt{1\times 1} + 4\sqrt{1\times 2} + 1\sqrt{2\times 2} = 8.8284$$
- $$H({\mathcal {G}}) = 2\left( \frac{2}{1+3}\right) + 4\left( \frac{2}{2+2}\right) + 4\left( \frac{2}{2+3}\right) + 1\left( \frac{2}{3+3}\right) = 4.1333$$
- $$SC({\mathcal {G}}) = 2 \left( \frac{1}{\sqrt{1+3}}\right) + 4 \left( \frac{1}{\sqrt{2+2}}\right) + 4 \left( \frac{1}{\sqrt{2+3}}\right) + 1 \left( \frac{1}{\sqrt{3+3}}\right) = 4.3027$$
- $$GA({\mathcal {G}}) = 2 \left( \frac{2\sqrt{1\times 3}}{1+3}\right) + 4 \left( \frac{2\sqrt{2\times 2}}{2+2}\right) +4 \left( \frac{2\sqrt{2\times 3}}{2+3}\right) + 1 \left( \frac{2\sqrt{3\times 3}}{3+3}\right) = 8.6916$$
- $$IS({\mathcal {G}}) = 2 \left( \frac{1\times 3}{1+3}\right) + 4 \left( \frac{2\times 2}{2+2}\right) + 4 \left( \frac{2\times 3}{2+3}\right) + 1 \left( \frac{3\times 3}{3+3}\right) = 9.4$$
- $$F({\mathcal {G}}) = 2(1^2 + 3^2) + 4(2^2 + 2^2) + 4(2^2 + 3^2) + 1(3^2 + 3^2) = 96$$
- $$SDI({\mathcal {G}}) = 2 \left( \frac{1^3+3^2}{1\times 3}\right) + 4 \left( \frac{2^3+2^2}{2\times 2}\right) + 4 \left( \frac{2^3+3^2}{2\times 3}\right) + 1 \left( \frac{3^3+3^2}{3\times 3}\right) = 21$$
- $$ABC({\mathcal {G}}) = 2\sqrt{\frac{1+3-2}{1\times 3}} + 4\sqrt{\frac{2+2-2}{2\times 2}} + 4\sqrt{\frac{2+3-2}{2\times 3}} + 1\sqrt{\frac{3+3-2}{3\times 3}} = 6.5423$$
- $$So({\mathcal {G}}) = 2\sqrt{1^2+3^2} + 4\sqrt{2^2+2^2} + 4\sqrt{2^2+3^2} + 1\sqrt{3^2+3^2} = 29.0926$$

$$\square$$

We determine the degree-based topological indices of each of the chosen compounds using MATLAB and a methodology similar to that in the previously discussed Theorem. The compounds’ computed indices are shown in the Tables 4 and 5.

**Table 4:** Calculated topological indices of the compounds (Part I).

Compound	${M_1}$	${M_2}$	${RM_2}$	HM	AZ	R	RR	RRR
Acetic acid	12	9	0	48	10.125	1.7321	5.1962	0
Ascorbic acid	58	68	22	292	91.0625	5.5746	27.6909	12.2426
Ascorbyl Palmitate	128	139	40	582	222.4375	13.9684	62.3219	29.0711
Benzoic acid	40	43	12	182	66.1406	4.3045	19.3631	8.8284
BHA	64	72	21	326	91.7007	5.9477	29.8576	12.5206
BHT	84	96	28	452	103.4015	7.0317	38.1903	14.5558
Citric acid	58	62	16	292	68.4444	5.7764	26.4122	8.7420
Dimethyl Dicarbonate	34	34	8	150	54.75	4.2019	16.0905	5.6569
Ethyl Lauroyl Arginate	110	113	29	476	192.8906	13.0064	53.4536	28.4853
Methyl 4-hydroxybenzoate	50	55	16	234	82.1406	5.2364	24.1258	11.0711
Propyl 4-hydroxybenzoate	58	63	18	266	98.1406	6.2364	28.1258	13.0711
Propyl gallate (PG)	70	79	24	338	111.6719	7.0577	33.5899	15.0711
Sorbic acid	28	26	5	114	46.7500	3.7701	13.3278	4.4142
TBHQ	55	61	17	283	71.0757	5.0015	25.2767	9.6921

**Table 5:** Calculated topological indices of the compounds (Part II).

Compound	H	SC	GA	IS	F	SDI	ABC	So
Acetic acid	1.5	1.5	2.5981	2.25	30	10	2.4495	9.4868
Ascorbic acid	5.2	5.5520	11.3463	13.2667	156	30.3333	8.7611	42.6724
Ascorbyl Palmitate	13.5	13.9464	28.1719	30.4167	304	66	20.8913	92.6438
Benzoic acid	4.1333	4.3027	8.6916	9.4	96	21	6.5423	29.0926
BHA	5.4857	5.9413	12.0976	14.031	182	35.5	9.6765	47.8665
BHT	6.2381	7.0446	14.4307	17.5286	260	49	12.2819	63.9707
Citric acid	5.1524	5.5361	10.8311	12.081	168	35.6667	9.2389	44.2521
Dimethyl Dicarbonate	3.9333	3.9436	7.5369	7.6333	82	20.3333	5.8756	25.2189
Ethyl Lauroyl Arginate	12.5667	12.7462	25.2285	26.0333	250	519.3333	18.7819	79.7667
Methyl 4-hydroxybenzoate	5	5.2217	10.5738	11.6667	124	26	7.9565	36.4879
Propyl 4-hydroxybenzoate	6	6.2217	12.5738	13.6667	140	30	9.3707	42.1447
Propyl gallate (PG)	6.6667	7.0382	14.3059	16.1667	180	36.6667	10.9228	51.2977
Sorbic acid	3.5667	3.5246	6.6547	6.3667	62	17.333	5.1685	20.6515
TBHQ	4.519	4.9695	10.0612	11.7143	161	3	8.3717	41.5816

Tables 6, 7, 8, 9, 10 and 11 show the linear, quadratic, and cubic regression models that use different degree-based topological indices as predictors to predict the physicochemical properties of the food preservtaives (MW, MP, BP, pKa, VD, Log P, and LD50). All the regression models are developed using MATLAB progamming. Here we consider the following metrics for evaluation:

- R (regression coefficient): Measures the strength of the relationship between the actual value and value predicted by the model. The range of values for R is $-1$ to 1. When the R-value is higher than 0.9, it indicates a strong positive correlation and a strong influence of the predictor variable on the dependent parameter.
- $\hbox {R}^2$ (coefficient of determination): Indicates the proportion of the variance in the dependent variable the model can account for. A higher $\hbox {R}^2$ value denotes a better fit and implies that the majority of the variation is explained by the model.
- RMSE (Root Mean Square Error): Demonstrates average error in the predictions. A smaller RMSE indicates a better model.
- F-statistic: It evaluates the overall significance of the model. A low *p* value and a higher F-statistic indicate that the independent factors largely account for the variance in the dependent variable.
- *p* value: It evaluates the model’s statistical significance. It is suggested that the predictor is statistically significant if the *p* value is less than 0.05.

**Fig. 4:**
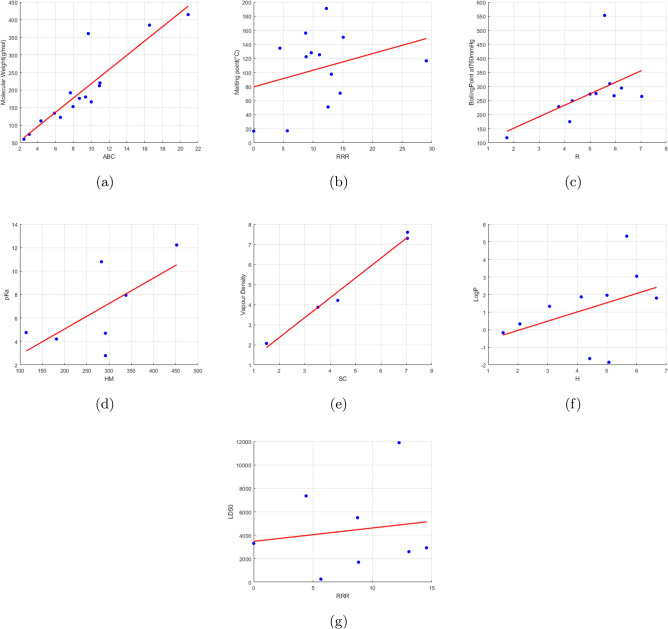
Linear regression plots.

In linear regression analysis, most of the computed indices show a positive correlation with the physicochemical properties. The Reduced Reciprocal Randić index shows the highest correlation with LD50 value and melting point. Vapour density and molecular weight have the strongest correlations with the Atom bond connectivity Index. Randic index, Hyper Zagreb index and Harmonic index shows a high association with boiling point, pKa value and Log P value respectively. Although they do not predominate, descriptors such as RM_2_, AZ, and GA exhibit reasonable relationships across many attributes. The regression coefficient between each property and index is displayed in Table 12. The linear regression plots of the physicochemical property and index pairs with the highest coefficient of determination are displayed in Fig. 4. These plots illustrate the strong predictive relationship between specific topological indices and selected physicochemical properties.

**Table 6:** Linear regression analysis.

Equation	R	${R^2}$	RMSE	F-statistic	*p* value
$MW = 3.1215M_1 + 16.9997$	0.9019	0.8135	44.3986	52.3308	0
$MP = 0.0485M_1 + 96.7147$	0.0266	0.0007	54.0850	0.0085	0.9280
$BP = 2.8670M_1 + 132.3403$	0.5211	0.2715	87.8094	3.3543	0.1003
$pKa = 0.1234 M_1 + 1.3941$	0.6406	0.4103	2.4482	4.1753	0.087
$VD = 0.0789 M_1 + 1.13185$	0.9876	0.9754	0.3332	118.7533	0.0017
$log~P = 0.0428 M_1 -0.8839$	0.4110	0.1689	1.9440	1.4228	0.2718
$LD50 = 30.3817 M_1 + 3437.6336$	0.1829	0.0334	3370.5546	0.2076	0.6647
$MW = 2.7738M_2 + 24.0218$	0.8896	0.7914	46.9468	45.5369	0.0000
$MP = 0.1268M_2 + 91.3234$	0.0773	0.0060	53.9424	0.0721	0.7929
$BP = 2.4808M_2 + 140.5099$	0.5540	0.3069	85.6467	3.9861	0.0770
$pKa = 0.0988M_2 + 0.6353$	0.6262	0.3921	2.6053	3.2246	0.1325
$VD = 0.0644M_2 + 1.7492$	0.9847	0.9696	0.3704	95.5742	0.0023
$log~P = 0.0336M_2 - 0.5726$	0.3938	0.1551	1.9601	1.2850	0.2943
$LD50 = 28.7151M_2 + 3409.3698$	0.2128	0.0453	3349.8183	0.2846	0.6129
$MW = 8.2326RM_2 + 45.7630$	0.7932	0.6292	62.5971	20.3630	0.0007
$MP = 1.3407RM_2 + 73.5128$	0.2454	0.0602	52.4491	0.7693	0.3977
$BP = 7.3567RM_2 + 152.9634$	0.5843	0.3415	83.4874	4.6665	0.0590
$pKa = 0.1409RM_2 + 3.9367$	0.2900	0.0841	3.1979	0.4589	0.5282
$VD = 0.1631RM_2 + 2.4010$	0.8649	0.7480	1.0657	8.9061	0.0584
$log~P = 0.0675RM_2 + 0.1114$	0.2766	0.0765	2.0493	0.5799	0.4712
$LD50 = 49.3466RM_2 + 4055.1278$	0.1350	0.0182	3396.9600	0.1114	0.7499
$MW = 0.6368HM + 26.5023$	0.8758	0.7670	53.5982	39.4997	0.0000
$MP = 0.0829HM + 83.6817$	0.2202	0.0485	54.2124	0.5603	0.4698
$BP = 0.4697HM + 164.1627$	0.4810	0.2313	99.7177	2.7086	0.1342
$pKa = 0.0260HM + 0.0980$	0.7680	0.5898	2.5321	7.1900	0.0437
$VD = 0.0133HM + 1.6271$	0.7582	0.5748	1.7942	5.4080	0.0806
$log~P = 0.0092HM - 0.7029$	0.5536	0.3065	2.2337	1.7676	0.2544
$LD50 = 0.1249HM + 4313.8638$	0.0041	0.0000	3972.8655	0.0001	0.9916
$MW = 0.4413AZ + 19.1789$	0.8901	0.7923	47.5157	50.0762	0
$MP = 0.1267AZ + 51.1888$	0.4063	0.1651	54.1512	2.3727	0.1479
$BP = 0.5065AZ + 102.2727$	0.5875	0.3452	95.1462	3.7175	0.0805
$pKa = 0.0112AZ + 2.1288$	0.5394	0.2909	2.5915	2.8629	0.1235
$VD = 0.0101AZ + 1.6421$	0.9872	0.9745	0.3411	152.0418	0.0003
$log~P = 0.0043AZ - 0.2049$	0.3287	0.1080	1.9427	0.9726	0.3487
$LD50 = 7.4644AZ + 2339.1877$	0.3365	0.1132	3095.1422	1.0211	0.3418
$MW = 3.6302R + 27.0927$	0.8764	0.7681	50.0842	46.2338	0
$MP = 1.0411R + 53.4704$	0.4373	0.1912	53.2048	2.8042	0.1266
$BP = 4.6839R + 57.1714$	0.6982	0.4875	87.1746	9.5063	0.0115
$pKa = 0.1512R + 2.3106$	0.5874	0.3450	2.5132	3.7121	0.0807
$VD = 0.0935R + 1.3176$	0.9845	0.9693	0.3676	125.2836	0.0004
$log~P = 0.0336R - 0.2764$	0.3461	0.1198	1.9024	1.0843	0.3238
$LD50 = 63.3421R + 2315.0936$	0.3744	0.1402	3050.1256	1.1392	0.3125
$MW = 2.2077RR + 25.3483$	0.8921	0.7969	46.6978	50.2742	0
$MP = 0.6332RR + 51.8729$	0.4312	0.1859	53.3772	2.7131	0.1317
$BP = 2.8363RR + 62.4172$	0.7108	0.5052	85.1373	10.3018	0.0101
$pK_a = 0.0946RR + 2.1368$	0.5932	0.3519	2.4874	3.8476	0.0781
$VD = 0.0535RR + 1.3694$	0.9886	0.9773	0.3216	169.4287	0.0002
$log~P = 0.0192RR - 0.2324$	0.3567	0.1272	1.8953	1.1433	0.3106
$LD50 = 36.4138RR + 2341.2246$	0.3842	0.1476	3031.2456	1.1986	0.3001
$MW = 5.2432RRR + 23.7485$	0.9024	0.8144	44.7321	55.1387	0
$MP = 1.4102RRR + 49.9362$	0.4378	0.1917	53.1954	2.7934	0.1268
$BP = 6.7204RRR + 55.8326$	0.7172	0.5144	83.8572	10.7691	0.0092
$pK_a = 0.2253RRR + 2.0413$	0.5771	0.3331	2.5107	3.7461	0.0821
$VD = 0.1277RRR + 1.1948$	0.9898	0.9797	0.3079	180.6244	0.0002
$log~P = 0.0458RRR - 0.3214$	0.3723	0.1386	1.8731	1.2298	0.2943
$LD50= 86.9285RRR + 2289.0543$	0.4086	0.1670	2995.3287	1.3315	0.2745

**Table 7:** Linear regression analysis.

Equation	R	${R^2}$	RMSE	F-statistic	*p* value
$MW = 28.8813\,H + 34.1201$	0.8840	0.7814	51.9136	42.8963	0.0000
$MP = 3.5946\,H + 84.9419$	0.2294	0.0526	51.8268	0.6667	0.4301
$BP = -2.0716\,H + 269.5216$	0.0677	0.0046	103.8197	0.0552	0.8182
$pKa = 1.4706\,H - 0.6778$	0.4519	0.2042	3.5270	1.2828	0.3088
$VD = 1.2035\,H - 0.1502$	0.9836	0.9674	0.4950	88.9777	0.0025
$log~P = 0.6057\,H - 1.2378$	0.4853	0.2355	1.9948	1.8484	0.2228
$LD50 = 586.4762\,H + 2274.4149$	0.2288	0.0523	3853.7237	0.3314	0.5858
$MW = 28.4983 \, SC + 28.2069$	0.8940	0.799	49.761	47.749	0.00001
$MP = 4.8039 \, SC + 78.2419$	0.2608	0.068	53.653	0.803	0.389
$BP = 39.3119 \, SC + 81.2253$	0.5536	0.306	94.717	3.978	0.077
$pK_a = 1.4407 \, SC - 1.0390$	0.5238	0.274	3.368	1.891	0.228
$VD = 0.9890 \, SC + 0.3793$	0.9936	0.987	0.310	230.76	0.0006
$log~P = 0.5103\, SC - 1.3096$	0.4011	0.161	2.215	1.342	0.285
$LD_{50} = 247.1597 \, SC + 3282.5675$	0.1165	0.0136	3462.3901	0.0825	0.7836
$MW = 14.0558 \, GA + 29.1268$	0.8935	0.7984	46.1575	47.5214	0.0000
$MP = 2.4617 \, GA + 77.2222$	0.2757	0.0760	49.1407	0.9052	0.3618
$BP = 17.8308 \, GA + 97.0457$	0.5532	0.3060	85.7052	3.9684	0.0775
$pKa = 0.8118\, GA-2.2340$	0.6499	0.4223	2.5397	3.6553	0.1141
$VD = 0.4587\, GA + 0.7540$	0.9914	0.9828	0.2782	171.6443	0.0010
$log~P = 0.2408 \,GA -1.1581$	0.4071	0.1657	1.9477	1.3906	0.2768
$LD50 = 110.9857\,GA + 3413.1774$	0.1129	0.0127	3463.8341	0.0774	0.7901
$MW = 12.9273 \, IS + 26.8941$	0.8904	0.7927	46.8009	45.8961	0.0000
$MP = 2.9674 \, IS + 67.4658$	0.3746	0.1403	47.4005	1.7953	0.2073
$BP = 15.1363 \, IS + 105.9877$	0.6058	0.3670	81.8504	5.2186	0.0482
$pKa = 0.0952 \,IS + 5.5712$	0.1019	0.0104	3.3240	0.0525	0.8279
$VD = 0.4078 \, IS + 1.0719$	0.9714	0.9437	0.5038	50.2757	0.0058
$log~P = 0.0298 \, IS + 0.9501$	0.0640	0.0041	2.1281	0.0288	0.8701
$LD50 = 166.5180 \,IS + 2692.1485$	0.2232	0.0498	3398.2034	0.3145	0.5953
$MW = 1.1868\, F + 20.2262$	0.8619	0.7429	52.1226	34.6773	0.0001
$MP = 0.1732 \,F + 79.9475$	0.2463	0.0606	49.5482	0.7101	0.4173
$BP = 0.7726 \,F + 170.7269$	0.4575	0.2093	91.4826	2.3821	0.1571
$pKa = 0.0380 \, F + 0.8985$	0.6654	0.4428	2.4942	3.9734	0.1028
$VD = 0.0242 \, F + 1.9672$	0.9559	0.9137	0.6238	31.7523	0.0111
$log~P = 0.0133 \, F - 0.4957$	0.3997	0.1598	1.9547	1.3311	0.2865
$LD50 = 6.1725 \, F + 3678.0637$	0.1203	0.0145	3460.7911	0.0881	0.7766
$MW = 6.0800 \, SDI + 2.5466$	0.8951	0.8012	45.8347	48.3627	0.0000
$MP = 0.8502 \, SDI + 79.0597$	0.2301	0.0529	49.7510	0.6149	0.4495
$BP = 4.3695 \, SDI + 151.3295$	0.4307	0.1855	92.8499	2.0493	0.1861
$pKa = 0.2186\, SDI - 0.1568$	0.6364	0.4050	2.5774	3.4037	0.1244
$VD = 0.1455 \,SDI + 1.1094$	0.9674	0.9359	0.5373	43.8302	0.0070
$log~P= 0.0775 \,SDI - 0.9091$	0.3969	0.1575	1.9573	1.3091	0.2902
$LD50 = 29.3959 \, SDI + 3659.8869$	0.0962	0.0093	3469.9437	0.0561	0.8207
$MW = 19.4254 \, ABC + 17.1923$	0.9028	0.8151	44.2025	52.9029	0.0000
$MP = 3.1310 \, ABC + 77.5602$	0.2584	0.0668	49.3861	0.7871	0.3940
$BP = 20.9745 \, ABC + 109.9478$	0.5152	0.2655	88.1719	3.2529	0.1048
$pKa = 0.8841 \, ABC - 0.9653$	0.5940	0.3528	2.6881	2.7259	0.1596
$VD = 0.5794 \,ABC + 0.6800$	0.9949	0.9898	0.2146	290.5792	0.0004
$log~P = 0.3098 \, ABC - 1.2071$	0.4103	0.1684	1.9447	1.4171	0.2727
$LD50 = 135.9916 \, ABC + 3430.3584$	0.1116	0.0125	3464.3409	0.0757	0.7925
$MW = 4.2984 \, So + 13.7476$	0.8998	0.8096	44.8602	51.0137	0.0000
$MP = 0.6722 \, So + 77.5588$	0.2621	0.0687	49.3354	0.8114	0.3870
$BP = 3.6400 \, So + 139.8451$	0.5007	0.2507	89.0574	3.0104	0.1168
$pKa = 0.1627\,So - 0.0480$	0.6369	0.4056	2.5761	3.4119	0.1240
$VD = 0.1044 \, So + 1.3663$	0.9827	0.9657	0.3931	84.4840	0.0027
$log~P = 0.0567 \, So - 0.8470$	0.4086	0.1669	1.9464	1.4026	0.2749
$LD50 = 26.7970 \, So + 3515.5127$	0.1222	0.0149	3459.9941	0.0909	0.7732

**Table 8:** Quadratic regression analysis.

Equation	R	${R^2}$	RMSE	F-statistic	*p* value
$MW = 0.0019 \,{M_1}^2 + 2.8451\, M_1 + 24.9104$	0.9022	0.8139	44.3444	24.0571	0.0001
$MP = -0.0166 \, {M_1}^2 + 2.8140 \, M_1 + 11.8139$	0.4622	0.2136	45.3338	1.3584	0.3007
$BP = -0.0856 \, {M_1}^2 + 10.9020 \, M_1 - 25.7457$	0.6446	0.4155	78.6519	2.8438	0.1167
$pKa = 0.0033 \, {M_1}^2 - 0.2425 \, M_1 + 9.0636$	0.7061	0.4985	2.3662	1.9883	0.2515
$VD = -0.0003 \, {M_1}^2 + 0.1070 \, M_1 + 0.8359$	0.9899	0.9799	0.3010	48.7421	0.0201
$log~P = 0.0016\, {M_1}^2 - 0.1090 \, M_1 + 2.0047$	0.5513	0.3039	1.7792	1.3097	0.3373
$LD50 = -1.2601 \, {M_1}^2 + 141.2059 \, M_1 + 1160.1080$	0.2221	0.0493	3399.0454	0.1297	0.8812
$MW = 0.0045 \, {M_2}^2 + 2.1047\, M_2 + 43.4145$	0.8919	0.7954	46.4962	21.3847	0.0002
$MP = -0.0132 \, {M_2}^2 + 2.3939 \, M_2 + 21.1674$	0.4787	0.2291	44.8847	1.4863	0.2722
$BP = -0.0540 \, {M_2}^2 + 7.9864 \, M_2 + 29.1706$	0.6551	0.4292	77.7285	3.0074	0.1062
$pKa = 0.0022 \, {M_2}^2 - 0.1691 \, M_2 + 7.7376$	0.7104	0.5047	2.3516	2.0379	0.2453
$VD = -0.0002 \, {M_2}^2 + 0.0860 \, M_2 + 1.3880$	0.9870	0.9742	0.3409	37.7717	0.0258
$log~P = 0.0011 \, {M_2}^2 - 0.0766 \, M_2 + 1.5619$	0.5323	0.2834	1.8052	1.1863	0.3680
$LD50 = -0.6589 \, {M_2}^2 + 90.0920 \, M_2 + 2022.5267$	0.2122	0.0450	3406.7461	0.1178	0.8912
$MW = 0.0752 \, {RM_2}^2 + 5.8794 \, RM_2 + 66.1071$	0.8607	0.7409	52.3281	15.7261	0.0006
$MP = -0.1324 \,{RM_2}^2 + 6.8227 \, RM_2 + 40.1129$	0.4913	0.2413	44.5283	1.5906	0.2513
$BP = -0.3295 \, {RM_2}^2 + 17.3202 \, RM_2 + 108.5572$	0.8630	0.4396	77.0152	3.1378	0.0986
$pKa = 0.0199 \, {RM_2}^2 - 0.3870 \, RM_2 + 6.3464$	0.6676	0.4457	2.4876	1.6085	0.3072
$VD = -0.0003 \, {RM_2}^2 + 0.2039 \, RM_2 + 2.2966$	0.9840	0.9683	0.3782	30.5181	0.0317
$log~P = 0.0082 \, {RM_2}^2 - 0.1358 \, RM_2 + 0.8314$	0.4546	0.2067	1.8994	0.7815	0.4993
$LD50 = 0.4474 \, {RM_2}^2 + 77.2225 \, RM_2 + 3276.4585$	0.2221	0.0493	3399.0239	0.1298	0.8812
$MW = 0.0003 \, {HM}^2 + 0.4594 \, HM + 43.3270$	0.8817	0.7774	48.4989	19.2101	0.0003
$MP = -0.0008 \, {HM}^2 + 0.6201 \, HM + 14.0399$	0.4883	0.2384	44.6139	1.5653	0.2562
$BP = -0.0030\, {HM}^2 + 1.9510 \, HM + 13.7672$	0.6616	0.4377	77.1479	3.1132	0.1000
$pKa = 0.0001 \, {HM}^2 - 0.0257 \, HM + 6.4734$	0.7223	0.5218	2.3107	2.1821	0.2287
$VD = -0.0000 \, {HM}^2 + 0.0254 \, HM + 0.9228$	0.9858	0.9717	0.3569	34.3884	0.0283
$log~P = 0.0001 \, {HM}^2 - 0.0172 \, HM + 1.7993$	0.5480	0.3003	1.7838	1.2875	0.3426
$LD50 = -0.0449 \, {HM}^2 + 26.2364 \, HM + 1452.2514$	0.2479	0.0615	3377.2959	0.1637	0.8534
$MW = -0.0012 \, {AZ}^2 + 2.0200 \, AZ + 31.4124$	0.8899	0.7920	46.8887	20.9364	0.0002
$MP = -0.0052 \, {AZ}^2 + 1.5584 \, AZ + 21.8408$	0.4693	0.2202	45.1444	1.4119	0.2883
$BP = -0.0122 \, {AZ}^2 + 3.8887 \, AZ + 66.0767$	0.6223	0.3872	80.5331	2.5278	0.1410
$pKa = 0.0005 \, {AZ}^2 - 0.0010 \, AZ + 3.4401$	0.5134	0.2636	2.8674	0.7160	0.5423
$VD = 0.0002 \, {AZ}^2 + 0.0288 \, AZ + 1.7865$	0.9847	0.9696	0.3703	31.8779	0.0304
$log\,P = 0.0005 \, {AZ}^2 - 0.0293 \, AZ + 0.4148$	0.4609	0.2124	1.8925	0.8091	0.4885
$LD50 = -0.0916 \, {AZ}^2 + 29.9503 \, AZ + 2919.6011$	0.1600	0.0256	3441.1868	0.0657	0.9372
$MW = -0.8931 \, {R}^2 + 43.6031 \, R - 25.2426$	0.8972	0.8049	45.4067	22.6903	0.0001
$MP = -1.6404 \, {R}^2 + 31.6531 \, R - 10.7942$	0.4580	0.2098	45.4456	1.3272	0.3082
$BP = -9.8512 \, {R}^2 + 127.2774 \, R - 97.5965$	0.5949	0.3539	82.6973	2.1906	0.1743
$pKa = 0.6425 \, {R}^2 - 5.6784 \, R + 17.6937$	0.5418	0.2936	2.8084	0.8312	0.4990
$VD = 0.0538 \, {R}^2 + 0.5374 \, R + 0.9884$	0.9984	0.9968	0.1199	312.2868	0.0032
$log\,P = 0.2009 \, {R}^2 - 1.3113 \, R + 2.1716$	0.4660	0.2172	1.8868	0.8322	0.4797
$LD50 = -194.4701 \, {R}^2 + 1974.9132 \, R - 78.8390$	0.1922	0.0370	3421.0900	0.0959	0.9102
$MW = 0.0027 \, {RR}^2 + 6.2244 \, RR + 24.3385$	0.9024	0.8143	44.3045	24.1103	0.0001
$MP = -0.0681 \, {RR}^2 + 5.6865 \, RR + 14.1401$	0.4653	0.2165	45.2515	1.3816	0.2953
$BP = -0.3575 \, {RR}^2 + 21.7422 \, RR - 11.3983$	0.6387	0.4080	79.1602	2.7562	0.1229
$pKa = 0.0170 \, {RR}^2 - 0.6115 \, RR + 10.0971$	0.6918	0.4786	2.4127	1.8361	0.2718
$VD = -0.0002 \, {RR}^2 + 0.1781 \, RR + 1.2092$	0.9918	0.9837	0.2715	60.1651	0.0163
$log\,P = 0.0075 \, {RR}^2 - 0.2318 \, RR + 1.8412$	0.5447	0.2967	1.7883	1.2659	0.3478
$LD50 = -4.9998 \, {RR}^2 + 264.4558 \, RR + 1525.1238$	0.2028	0.0411	3413.6492	0.1073	0.9003
$MW = 0.0008 \, {RRR}^2 + 11.7487 \, RRR + 60.5884$	0.8910	0.7939	46.6689	21.1861	0.0002
$MP = -0.2568 \, {RRR}^2 + 9.8904 \, RRR + 38.7436$	0.4775	0.2280	44.9183	1.4766	0.2742
$BP = -0.8348 \, {RRR}^2 + 27.6779 \, RRR + 104.0943$	0.6277	0.3940	80.0849	2.6011	0.1348
$pKa = 0.0504 \, {RRR}^2 - 0.4920 \, RRR + 5.7712$	0.5752	0.3309	2.7332	0.9891	0.4477
$VD = 0.0106 \, {RRR}^2 + 0.1876 \, RRR + 2.2640$	0.9816	0.9636	0.4051	26.4614	0.0364
$log\,P = 0.0281 \, {RRR}^2 - 0.2471 \, RRR + 0.4094$	0.5030	0.2531	1.8430	1.0163	0.4167
$LD50 = -5.9482 \, {RRR}^2 + 203.9391 \, RRR + 3273.0388$	0.1545	0.0239	3444.2302	0.0612	0.9414

**Table 9:** Quadratic regression analysis.

Equation	R-value	${R^2}$	RMSE	F-statistic	*p* value
$MW = -1.0316 \, H^2 + 45.6094 \, H - 18.7303$	0.8907	0.7934	46.7232	21.1240	0.0002
$MP = -1.7949 \, H^2 + 33.2807 \, H - 9.6039$	0.4683	0.2193	45.1713	1.4043	0.2901
$BP = -8.6709 \, H^2 + 113.9993 \, H - 54.1021$	0.5878	0.3455	83.2326	2.1112	0.1835
$pKa = 0.4520 \, H^2 - 3.1916 \, H + 10.8655$	0.4698	0.2207	2.9497	0.5664	0.6073
$VD = 0.0756 \, H^2 + 0.4768 \, H + 1.1563$	0.9893	0.9786	0.3102	45.8277	0.0214
$log\,P = 0.1942 \, H^2 - 1.0515 \, H + 1.4104$	0.4487	0.2014	1.9057	0.7565	0.5094
$LD50 = -212.1748 \, H^2 + 1952.0651 \, H + 396.6777$	0.1805	0.0326	3428.8522	0.0842	0.9205
$MW = -0.7205 \, SC^2 + 40.3754 \, SC - 10.3874$	0.8980	0.8064	45.2267	22.9152	0.0001
$MP = -1.5712 \, SC^2 + 30.0561 \, SC - 2.9061$	0.4635	0.2149	45.2989	1.3683	0.2984
$BP = -9.1247 \, SC^2 + 117.3429 \, SC - 63.0340$	0.6028	0.3633	82.0902	2.2825	0.1643
$pKa = 0.5698 \, SC^2 - 4.7469 \, SC + 14.9171$	0.5733	0.3287	2.7377	0.9794	0.4506
$VD = 0.0536 \, SC^2 + 0.5054 \, SC + 1.2264$	0.9974	0.9948	0.1524	193.0910	0.0052
$log\,P = 0.2044 \, SC^2 - 1.2920 \, SC + 1.9900$	0.4979	0.2479	1.8493	0.9889	0.4254
$LD50 = -175.9982 \, SC^2 + 1760.1808 \, SC + 534.5749$	0.1907	0.0364	3422.1640	0.0943	0.9116
$MW = -0.0981 \, GA^2 + 17.2411 \, GA + 8.7495$	0.8950	0.8010	45.8624	22.1329	0.0001
$MP = -0.3545 \, GA^2 + 13.6932 \, GA + 6.1677$	0.4677	0.2188	45.1862	1.4001	0.2910
$BP = -2.1570 \, GA^2 + 54.4934 \, GA - 32.6038$	0.6057	0.3668	81.8638	2.3173	0.1607
$pKa = 0.1513 \, GA^2 - 2.3974 \, GA + 13.6596$	0.7258	0.5268	2.2985	2.2266	0.2239
$VD = 0.0109 \, GA^2 + 0.2622 \, GA + 1.4072$	0.9945	0.9889	0.2232	89.4665	0.0111
$log\,P = 0.0491 \, GA^2 - 0.6196 \, GA + 1.8689$	0.5159	0.2661	1.8268	1.0879	0.3952
$LD50 = -34.1036 \, GA^2 + 694.6701 \, GA + 1363.1084$	0.1733	0.0300	3433.3551	0.0774	0.9266
$MW = 0.0148 \, IS^2 + 12.4298 \, IS + 30.1966$	0.8904	0.7928	46.7905	21.0475	0.0002
$MP = -0.2908 \, IS^2 + 12.5162 \, IS + 4.7003$	0.5403	0.2920	43.0172	2.0617	0.1779
$BP = -0.9773 \, IS^2 + 34.2528 \, IS + 30.6710$	0.6340	0.4019	79.5616	2.6882	0.1279
$pKa = -0.1485 \, IS^2 + 3.6528 \, IS - 13.7701$	0.5632	0.3172	2.7610	0.9292	0.4662
$VD = 0.0032 \, IS^2 + 0.3473 \, IS + 1.2717$	0.9719	0.9446	0.4996	17.0555	0.0554
$log\,P = -0.0433 \, IS^2 + 0.8876 \, IS - 2.2674$	0.4339	0.1883	1.9212	0.6959	0.5348
$LD50 = 11.3039 \, IS^2 - 56.9570 \, IS + 3545.2729$	0.2338	0.0546	3389.5317	0.1445	0.8689
$MW = 0.0008 F^2 + 0.9072 F + 38.7799$	0.8635	0.7457	51.8433	16.1249	0.0005
$MP = -0.0034 F^2 + 1.3012 F + 5.2699$	0.5069	0.2569	44.0682	1.7289	0.2265
$BP = -0.0103 F^2 + 3.6559 F + 6.7831$	0.6625	0.4390	77.0595	3.1296	0.0991
$pKa = 0.0002 F^2 - 0.0313 F + 5.6409$	0.7207	0.5194	2.3165	2.1612	0.2310
$VD = -0.0001 F^2 + 0.0539 F + 0.5579$	0.9867	0.9736	0.3449	36.8963	0.0264
$log~P = 0.0002 F^2 - 0.0312 F + 1.9901$	0.5528	0.3056	1.7770	1.3203	0.3348
$LD50 = -0.1684 F^2 + 54.6082 F + 1037.3817$	0.2778	0.0772	3348.8623	0.2091	0.8180
$MW = 0.0054\, SDI^2 + 5.6586\, SDI + 9.3079$	0.8952	0.8014	45.8124	22.1932	0.0001
$MP = -0.0727\, SDI^2 + 6.3430\, SDI - 7.8831$	0.4445	0.1976	45.7952	1.2310	0.3327
$BP = -0.3621\, SDI^2 + 25.4357\, SDI - 117.3140$	0.6440	0.4148	78.7032	2.8349	0.1173
$pKa = 0.0078\, SDI^2 - 0.2868\, SDI + 7.3345$	0.6839	0.4677	2.4379	1.7573	0.2833
$VD = -0.0033\, SDI^2 + 0.3439\, SDI - 1.1603$	0.9937	0.9875	0.2378	78.6844	0.0125
$log~P = 0.0052\, SDI^2 - 0.2287\, SDI + 2.9275$	0.5340	0.2852	1.8029	1.1970	0.3652
$LD50 = -6.2902\, SDI^2 + 401.3109\, SDI - 967.4484$	0.2819	0.0795	3344.7401	0.2158	0.8130
$MW = -0.1614\, ABC^2 + 23.3581\, ABC - 2.1152$	0.9038	0.8170	43.9735	24.5578	0.0001
$MP = -0.6688\, ABC^2 + 19.0173\, ABC + 0.5181$	0.4533	0.2055	45.5676	1.2934	0.3165
$BP = -4.3526\, ABC^2 + 84.2122\, ABC - 90.7404$	0.6266	0.3927	80.1759	2.5861	0.1361
$pKa = 0.1966\, ABC^2 - 2.5423\, ABC + 12.9731$	0.6678	0.4459	2.4872	1.6097	0.3070
$VD = -0.0022\, ABC^2 + 0.6127\, ABC + 0.5834$	0.9949	0.9899	0.2136	97.8067	0.0101
$log~P = 0.0821\, ABC^2 - 0.8958\, ABC + 2.5226$	0.5382	0.2897	1.7973	1.2234	0.3584
$LD50 = -72.0513\, ABC^2 + 1193.6939\, ABC + 139.3118$	0.2223	0.0494	3398.8996	0.1299	0.8810
$MW = 0.0056 \, So^2 + 3.7197 \, So + 25.8510$	0.9003	0.8106	44.7418	23.5344	0.0001
$MP = -0.0325 \, So^2 + 3.9712 \, So + 9.2657$	0.4618	0.2132	45.3456	1.3551	0.3015
$BP = -0.1609 \, So^2 + 15.1854 \, So - 34.9961$	0.6483	0.4203	78.3304	2.9001	0.1129
$pKa = 0.0053 \, So^2 - 0.2782 \, So + 8.2752$	0.7084	0.5019	2.3583	2.0152	0.2481
$VD = -0.0008 \, So^2 + 0.1675 \, So + 0.5327$	0.9892	0.9786	0.3109	45.6368	0.0214
$log\,P = 0.0028 \, So^2 - 0.1449 \, So + 2.1155$	0.5504	0.3030	1.7803	1.3040	0.3386
$LD50 = -2.4443 \, So^2 + 204.9751 \, So + 893.5669$	0.2364	0.0559	3387.3384	0.1479	0.8661

**Table 10:** Cubic regression analysis.

Equation	R	${R^2}$	RMSE	F-statistic	*p* value
$MW = -0.0000\, {M_1}^3 + 0.0047\, {M_1}^2 + 2.6889\, {M_1} + 27.1678$	0.9022	0.8139	44.3423	14.5817	0.0006
$MP = 0.0006\, {M_1}^3 - 0.1384\, {M_1}^2 + 9.6013\, {M_1} - 84.1763$	0.5681	0.3227	42.0728	1.4294	0.2973
$BP = -0.0023\, {M_1}^3 + 0.2485\, {M_1}^2 - 3.2876\, {M_1} + 131.0060$	0.6759	0.4569	75.8189	1.9628	0.2082
$pKa = 0.0001\, {M_1}^3 - 0.0120\, {M_1}^2 + 0.5601\, {M_1} - 3.7611$	0.7142	0.5100	2.3390	1.0409	0.4873
$VD = -0.0000\, {M_1}^3 + 0.0015\, {M_1}^2 + 0.0368\, {M_1} + 1.5262$	0.9918	0.9837	0.2711	20.1015	0.1622
$log~P = 0.0001\, {M_1}^3 - 0.0154\, {M_1}^2 + 0.6058\, {M_1} - 5.5482$	0.7385	0.5454	1.4378	1.9995	0.2327
$LD50 = -0.1187\, {M_1}^3 + 16.0148\, {M_1}^2 - 576.5868\, {M_1} + 8912.1186$	0.4046	0.1637	3188.0429	0.2610	0.8507
$MW = -0.0000\, {M_2}^3 + 0.0126\, {M_2}^2 + 1.6275\, {M_2} + 50.1580$	0.8920	0.7957	46.4641	12.9829	0.0009
$MP = 0.0003\, {M_2}^3 - 0.0878\, {M_2}^2 + 6.7525\, {M_2} - 38.6074$	0.5573	0.3106	42.4484	1.3513	0.3183
$BP = -0.0015\, {M_2}^3 + 0.1859\, {M_2}^2 - 2.8606\, {M_2} + 145.1459$	0.6949	0.4829	73.9811	2.1789	0.1785
$pKa = 0.0000\, {M_2}^3 - 0.0047\, {M_2}^2 + 0.2174\, {M_2} + 1.4256$	0.7160	0.5127	2.3325	1.0521	0.4838
$VD = -0.0000\, {M_2}^3 + 0.0006\, {M_2}^2 + 0.0520\, {M_2} + 1.7010$	0.9881	0.9763	0.3270	13.7198	0.1953
$log~P = 0.0001\, {M_2}^3 - 0.0101\, {M_2}^2 + 0.4242\, {M_2} - 3.4182$	0.7380	0.5446	1.4390	1.9933	0.2336
$LD50 = -0.0807\, {M_2}^3 + 12.2368\, {M_2}^2 - 478.0736\, {M_2} + 7934.0899$	0.4538	0.2059	3106.5286	0.3457	0.7953
$MW = -0.0017\, {RM_2}^3 + 0.1773\, {RM_2}^2 + 4.3094\, {RM_2} + 70.4378$	0.8611	0.7414	52.2726	9.5583	0.0028
$MP = 0.0074\, {RM_2}^3 - 0.5785\, {RM_2}^2 + 13.5355\, {RM_2} + 22.4997$	0.5286	0.2794	43.3958	1.1634	0.3762
$BP = -0.0477\, {RM_2}^3 + 1.7262\, {RM_2}^2 - 5.4672\, {RM_2} + 146.7865$	0.9071	0.8999	72.7515	2.3327	0.0005
$pKa = 0.0019\, {RM_2}^3 - 0.0753\, {RM_2}^2 + 0.9692\, {RM_2} + 1.3049$	0.6896	0.4756	2.4197	0.9069	0.5311
$VD = 0.0001\, {RM_2}^3 - 0.0036\, {RM_2}^2 + 0.2339\, {RM_2} + 2.2651$	0.9842	0.9687	0.3758	10.3027	0.2242
$log~P = 0.0022\, {RM_2}^3 - 0.0875\, {RM_2}^2 + 0.9000\, {RM_2} - 0.5002$	0.6887	0.4743	1.5462	1.5035	0.3214
$LD50 = -2.4985\, {RM_2}^3 + 106.2938\, {RM_2}^2 - 1044.2074\, {RM_2} + 5043.5035$	0.4656	0.2167	3085.2781	0.3690	0.7805
$MW = 0.0000\, {HM}^3 + 0.0001\, {HM}^2 + 0.5224\, {HM} + 39.3083$	0.8818	0.7775	48.4913	11.6472	0.0013
$MP = 0.0000\, {HM}^3 - 0.0052\, {HM}^2 + 1.7469\, {HM} - 56.4406$	0.5658	0.3201	42.1537	1.4124	0.3018
$BP = -0.0000\, {HM}^3 + 0.0050\, {HM}^2 + 0.2168\, {HM} + 106.3157$	0.6797	0.4620	75.4574	2.0041	0.2021
$pKa = -0.0000\, {HM}^3 + 0.0001\, {HM}^2 - 0.0306\, {HM} + 6.8342$	0.7224	0.5218	2.3107	1.0912	0.4723
$VD = -0.0000\, {HM}^3 + 0.0000\, {HM}^2 + 0.0119\, {HM} + 1.5720$	0.9888	0.9778	0.3164	14.6756	0.1890
$log~P = 0.0000\, {HM}^3 - 0.0005\, {HM}^2 + 0.1003\, {HM} - 4.1022$	0.7519	0.5654	1.4059	2.1680	0.2102
$LD50 = -0.0009\, {HM}^3 + 0.6677\, {HM}^2 - 123.2535\, {HM} + 9186.3619$	0.4951	0.2451	3028.8685	0.4330	0.7412
$MW = -0.0000\, {AZ}^3 + 0.0037\, {AZ}^2 + 1.6117\, {AZ} + 39.3221$	0.8903	0.7925	46.8217	12.7346	0.0009
$MP = 0.0001\, {AZ}^3 - 0.0327\, {AZ}^2 + 3.7978\, {AZ} - 18.7278$	0.5189	0.2693	43.7000	1.1057	0.3963
$BP = -0.0018\, {AZ}^3 + 0.2998\, {AZ}^2 - 10.8064\, {AZ} + 203.8246$	0.6790	0.4611	75.5263	1.9962	0.2033
$pKa = -0.0000\, {AZ}^3 + 0.0112\, {AZ}^2 - 0.7891\, {AZ} + 21.9078$	0.5223	0.2728	2.8495	0.3751	0.7790
$VD = -0.0000\, {AZ}^3 + 0.0006\, {AZ}^2 + 0.0130\, {AZ} + 1.9264$	0.9848	0.9699	0.3685	10.7289	0.2199
$log~P = 0.0000\, {AZ}^3 - 0.0025\, {AZ}^2 + 0.1196\, {AZ} - 1.0362$	0.4892	0.2393	1.8598	0.5244	0.6843
$LD50 = -0.0640\, {AZ}^3 + 10.8172\, {AZ}^2 - 468.7693\, {AZ} + 7395.1958$	0.3162	0.1000	3307.2395	0.1481	0.9257
$MW = -0.0324\, {R}^3 - 0.1774\, {R}^2 + 39.3328\, {R} - 18.3369$	0.8972	0.8050	45.3987	13.7577	0.0007
$MP = 0.5679\, {R}^3 - 14.3245\, {R}^2 + 106.9710\, {R} -130.7739$	0.5271	0.2779	43.4434	1.1543	0.3793
$BP = -10.3448\, {R}^3 + 126.6456\, {R}^2 - 416.5625\, {R} + 514.1967$	0.6779	0.4596	75.6282	1.9845	0.2050
$pKa = 2.0888\, {R}^3 - 33.4387\, {R}^2 + 174.7194\, {R} -291.6828$	0.7051	0.4972	2.3694	0.9888	0.5036
$VD = 0.0386\, {R}^3 - 0.4420\, {R}^2 + 2.3887\, {R} -0.9406$	0.9986	0.9973	0.1113	120.9979	0.0667
$log~P = 0.2878\, {R}^3 - 3.6907\, {R}^2 + 14.4678\, {R} -15.6684$	0.6634	0.4401	1.5957	1.3099	0.3685
$LD50 = -356.7353\, {R}^3 + 4468.2028\, {R}^2 - 16311.8112\, {R} + 20174.9542$	0.4017	0.1613	3192.5405	0.2565	0.8537
$MW = -0.0006\, {RR}^3 + 0.0576\, {RR}^2 + 4.7661\, {RR} + 34.1190$	0.9026	0.8146	44.2580	14.6500	0.0005
$MP = 0.0050\, {RR}^3 - 0.5617\, {RR}^2 + 18.6447\, {RR} -70.2282$	0.5608	0.3145	42.3260	1.3765	0.3114
$BP = -0.0275\, {RR}^3 + 1.4690\, {RR}^2 - 13.1462\, {RR} + 157.8832$	0.6827	0.4660	75.1783	2.0363	0.1975
$pKa = 0.0016\, {RR}^3 - 0.1103\, {RR}^2 + 2.4612\, {RR} -12.6405$	0.7131	0.5084	2.3427	1.0344	0.4892
$VD = -0.0000\, {RR}^3 + 0.0028\, {RR}^2 + 0.1284\, {RR} + 1.4171$	0.9920	0.9840	0.2686	20.4962	0.1606
$log~P = 0.0012\, {RR}^3 - 0.0737\, {RR}^2 + 1.3064\, {RR} -5.2697$	0.7361	0.5419	1.4433	1.9716	0.2367
$LD50 = -1.1598\, {RR}^3 + 71.1183\, {RR}^2 - 1157.0262\, {RR} + 8267.2737$	0.3845	0.1478	3218.1667	0.2313	0.8705

**Table 11:** Cubic regression analysis.

Equation	R	${R^2}$	RMSE	F-statistic	*p* value
$MW = -0.0284\, {H}^3 - 0.4363\, {H}^2 + 42.3192\, {H} - 13.8984$	0.8908	0.7935	46.7183	12.8058	0.0009
$MP = 0.5575\, {H}^3 - 13.5784\, {H}^2 + 97.9420\, {H} - 102.6588$	0.5202	0.2706	43.6604	1.1131	0.3937
$BP = -18.8349\, {H}^3 + 213.7755\, {H}^2 - 672.9522\, {H} + 712.0310$	0.6951	0.4831	73.9624	2.1812	0.1782
$pKa = 0.1610\, {H}^3 - 2.0035\, {H}^2 + 8.9766\, {H} - 8.7203$	0.4709	0.2218	2.9477	0.2850	0.8350
$VD = -0.1033\, {H}^3 + 1.3117\, {H}^2 - 3.8199\, {H} + 5.2171$	0.9940	0.9881	0.2315	27.6890	0.1386
$log~P = 0.2123\, {H}^3 - 2.4460\, {H}^2 + 8.7088\, {H} - 8.3615$	0.5289	0.2797	1.8098	0.6473	0.6176
$LD50 = -732.3403\, {H}^3 + 8368.5692\, {H}^2 - 27974.3427\, {H} + 29111.6779$	0.4675	0.2185	3081.7808	0.3728	0.7781
$MW = -0.0464\, {SC}^3 + 0.2924\, {SC}^2 + 34.4724\, {SC} - 1.2754$	0.8981	0.8066	45.2057	13.9040	0.0007
$MP = 0.5407\, {SC}^3 - 13.4859\, {SC}^2 + 98.8880\, {SC} - 106.6745$	0.5367	0.2880	43.1374	1.2135	0.3597
$BP = -8.6397\, {SC}^3 + 102.2196\, {SC}^2 - 309.8703\, {SC} + 383.9249$	0.6826	0.4659	75.1846	2.0356	0.1976
$pKa = 1.3384\, {SC}^3 - 20.7136\, {SC}^2 + 104.3328\, {SC} - 164.9056$	0.6711	0.4504	2.4771	0.8195	0.5630
$VD = 0.0684\, {SC}^3 - 0.8019\, {SC}^2 + 3.5552\, {SC} - 1.6896$	0.9991	0.9982	0.0900	185.2779	0.0539
$log~P = 0.2447\, {SC}^3 - 3.0317\, {SC}^2 + 11.3459\, {SC} - 11.2168$	0.6750	0.4557	1.5733	1.3953	0.3466
$LD50 = -293.8952\, {SC}^3 + 3573.0896\, {SC}^2 - 12364.7966\, {SC} + 15043.5091$	0.3920	0.1537	3207.1254	0.2421	0.8633
$MW = -0.0062\, {GA}^3 + 0.1709\, {GA}^2 + 14.1234\, {GA} + 17.9955$	0.8951	0.8012	45.8322	13.4360	0.0008
$MP = 0.0589\, {GA}^3 - 2.9587\, {GA}^2 + 43.5318\, {GA} - 79.5863$	0.5351	0.2863	43.1877	1.2037	0.3629
$BP = -0.6962\, {GA}^3 + 15.7190\, {GA}^2 - 80.6083\, {GA} + 237.1314$	0.6626	0.4391	77.0527	1.8263	0.2302
$pKa = -0.0454\, {GA}^3 + 1.5893\, {GA}^2 + -16.9039\, {GA} + 47.5869$	0.9419	0.8854	2.2406	1.2240	0.0060
$VD = 0.0094\, {GA}^3 - 0.2216\, {GA}^2 + 1.8685\, {GA} - 1.4546$	0.9998	0.9996	0.0437	786.3431	0.0262
$log~P = 0.0239\, {GA}^3 - 0.5837\, {GA}^2 + 4.2465\, {GA} - 7.7359$	0.6803	0.4628	1.5630	1.4356	0.3369
$LD50 = -25.6397\, {GA}^3 + 618.3987\, {GA}^2 - 4135.0799\, {GA} + 10780.7542$	0.3521	0.1240	3262.8268	0.1887	0.8990
$MW = -0.0015\, {IS}^3 + 0.0841\, {IS}^2 + 11.5529\, {IS} + 32.9282$	0.8904	0.7929	46.7865	12.7588	0.0009
$MP = 0.0072\, {IS}^3 - 0.6337\, {IS}^2 + 16.8010\, {IS} - 8.1856$	0.5427	0.2945	42.9390	1.2525	0.3474
$BP = -0.1780\, {IS}^3 + 4.3599\, {IS}^2 - 11.6831\, {IS} + 128.6868$	0.6531	0.4266	77.9033	1.7360	0.2464
$pKa = -0.0440\, {IS}^3 + 1.4357\, {IS}^2 - 14.2536\, {IS} + 48.5892$	0.7171	0.5143	2.3288	1.0587	0.4819
$VD = -0.0029\, {IS}^3 + 0.0824\, {IS}^2 - 0.2417\, {IS} + 2.3278$	0.9759	0.9523	0.4634	6.6616	0.2757
$log~P = -0.0059\, {IS}^3 + 0.1365\, {IS}^2 - 0.6573\, {IS} + 0.8784$	0.4970	0.2470	1.8504	0.5468	0.6716
$LD50 = -3.5414\, {IS}^3 + 115.9015\, {IS}^2 - 933.2465\, {IS} + 5368.7972$	0.2548	0.0649	3371.0144	0.0926	0.9602
$MW = 0.0000\, F^3 + 0.0001\, F^2 + 1.0085\, F + 35.2052$	0.8635	0.7457	51.8395	9.7746	0.0026
$MP = 0.0000\, F^3 - 0.0191\, F^2 + 3.4629\, F - 69.8912$	0.5669	0.3214	42.1138	1.4208	0.2996
$BP = -0.0000\, F^3 + 0.0009\, F^2 + 2.2612\, F + 51.1326$	0.6659	0.4435	76.7495	1.8592	0.2247
$pKa = -0.0000\, F^3 + 0.0007\, F^2 - 0.0981\, F + 8.3898$	0.7217	0.5209	2.3129	1.0871	0.4734
$VD = -0.0000\, F^3 + 0.0001\, F^2 + 0.0274\, F + 1.3268$	0.9897	0.9795	0.3038	15.9476	0.1816
$log~P= 0.0000\, F^3 - 0.0017\, F^2 + 0.1984\, F - 4.9002$	0.7677	0.5894	1.3665	2.3923	0.1847
$LD50 = -0.0055\, F^3 + 2.2249\, F^2 - 234.6687\, F + 9903.1487$	0.5101	0.2603	2998.3607	0.4691	0.7199
$MW = 0.0023\, SDI^3 - 0.2490\, SDI^2 + 13.8306\, SDI - 64.3043$	0.8987	0.8076	45.0872	13.9947	0.0007
$MP = 0.0053\, SDI^3 - 0.6702\, SDI^2 + 25.5254\, SDI - 179.8482$	0.5744	0.3300	41.8469	1.4773	0.2852
$BP = -0.0032\, SDI^3 - 0.0800\, SDI^2 + 17.9234\, SDI - 59.9520$	0.6455	0.4166	78.5783	1.6664	0.2598
$pKa = 0.0008\, SDI^3 - 0.0748\, SDI^2 + 2.2945\, SDI - 17.4628$	0.6987	0.4882	2.3904	0.9539	0.5150
$VD = -0.0001\, SDI^3 + 0.0064\, SDI^2 + 0.0958\, SDI + 0.6310$	0.9976	0.9951	0.1479	68.3555	0.0886
$log~P = 0.0008\, SDI^3 - 0.0629\, SDI^2 + 1.5726\, SDI - 10.5069$	0.7426	0.5514	1.4283	2.0486	0.2258
$LD50 = -0.7224\, SDI^3 + 57.6431\, SDI^2 - 1265.1715\, SDI + 11447.7437$	0.4125	0.1702	3175.6327	0.2735	0.8424
$MW = 0.0011\, ABC^3 - 0.1985\, ABC^2 + 23.7013\, ABC - 2.9687$	0.9039	0.8170	43.9733	14.8837	0.0005
$MP = 0.1732\, ABC^3 - 6.5450\, ABC^2 + 72.9398\, ABC - 131.1969$	0.5634	0.3175	42.2353	1.3954	0.3063
$BP = -0.9610\, ABC^3 + 17.1454\, ABC^2 - 58.9012\, ABC + 173.4715$	0.6649	0.4421	76.8411	1.8493	0.2263
$pKa = 0.1048\, ABC^3 - 2.5517\, ABC^2 + 20.3880\, ABC - 47.4516$	0.7154	0.5118	2.3346	1.0485	0.4849
$VD = -0.0043\, ABC^3 + 0.0874\, ABC^2 + 0.0733\, ABC + 1.4731$	0.9958	0.9917	0.1933	39.8914	0.1157
$log~P = 0.0470\, ABC^3 - 0.9691\, ABC^2 + 6.0563\, ABC - 9.9696$	0.7418	0.5502	1.4302	2.0388	0.2272
$LD50 = -39.3417\, ABC^3 + 799.6506\, ABC^2 - 4505.7665\, ABC + 10472.7816$	0.3680	0.1354	3241.5237	0.2088	0.8856
$MW = 0.0001\, So^3 - 0.0089\, So^2 + 4.3238\, So + 19.2954$	0.9004	0.8107	44.7272	14.2747	0.0006
$MP = 0.0016\, So^3 - 0.2687\, So^2 + 13.7276\, So - 94.7956$	0.5711	0.3261	41.9659	1.4520	0.2915
$BP = -0.0044\, So^3 + 0.3360\, So^2 - 0.9853\, So + 104.1762$	0.6698	0.4487	76.3899	1.8988	0.2182
$pKa = 0.0001\, So^3 - 0.0132\, So^2 + 0.4547\, So - 0.5111$	0.7125	0.5077	2.3446	1.0311	0.4902
$VD = -0.0000\, So^3 + 0.0031\, So^2 + 0.0495\, So + 1.4548$	0.9923	0.9846	0.2630	21.3801	0.1574
$log~P = 0.0003\, So^3 - 0.0274\, So^2 + 0.8288\, So - 5.9280$	0.7393	0.5466	1.4359	2.0091	0.2313
$LD50 = -0.2881\, So^3 + 29.6352\, So^2 - 816.5481\, So + 9481.1897$	0.4187	0.1753	3165.7712	0.2835	0.8358

**Table 12:** Linear regression coefficients of properties with molecular descriptors.

	M${_1}$	M${_2}$	RM${_2}$	HM	AZ	R	RR	RRR	H	SC	GA	IS	F	SDI	ABC	So
MW	0.9019	0.8896	0.7932	0.8758	0.8901	0.8764	0.8921	0.9024	0.8840	0.8940	0.8935	0.8904	0.8619	0.8951	**0.9028**	0.8998
MP	0.0266	0.0773	0.2454	0.2202	0.4063	0.4373	0.4312	**0.4378**	0.2294	0.2608	0.2757	0.3746	0.2463	0.2301	0.2584	0.2621
BP	0.5211	0.5540	0.5843	0.4810	0.5875	**0.6982**	0.7108	0.7172	0.0677	0.5536	0.5532	0.6058	0.4575	0.4307	0.5152	0.5007
pKa	0.6406	0.6262	0.2900	**0.7680**	0.5394	0.5874	0.5932	0.5771	0.4519	0.5238	0.6499	0.1019	0.6654	0.6364	0.5940	0.6369
VD	0.9876	0.9847	0.8649	0.7582	0.9872	0.9845	0.9886	0.9898	0.9836	**0.9936**	0.9914	0.9714	0.9559	0.9674	**0.9949**	0.9827
log P	0.4110	0.3938	0.2766	0.5536	0.3287	0.3461	0.3567	0.3723	**0.4853**	0.4011	0.4071	0.0640	0.3997	0.3964	0.4103	0.4086
LD50	0.1829	0.2128	0.1350	0.0041	0.3365	0.3744	0.3842	**0.4086**	0.2288	0.1165	0.1129	0.2232	0.1203	0.0964	0.1116	0.1222

Bold values indicate the highest correlation.

**Fig. 5:**
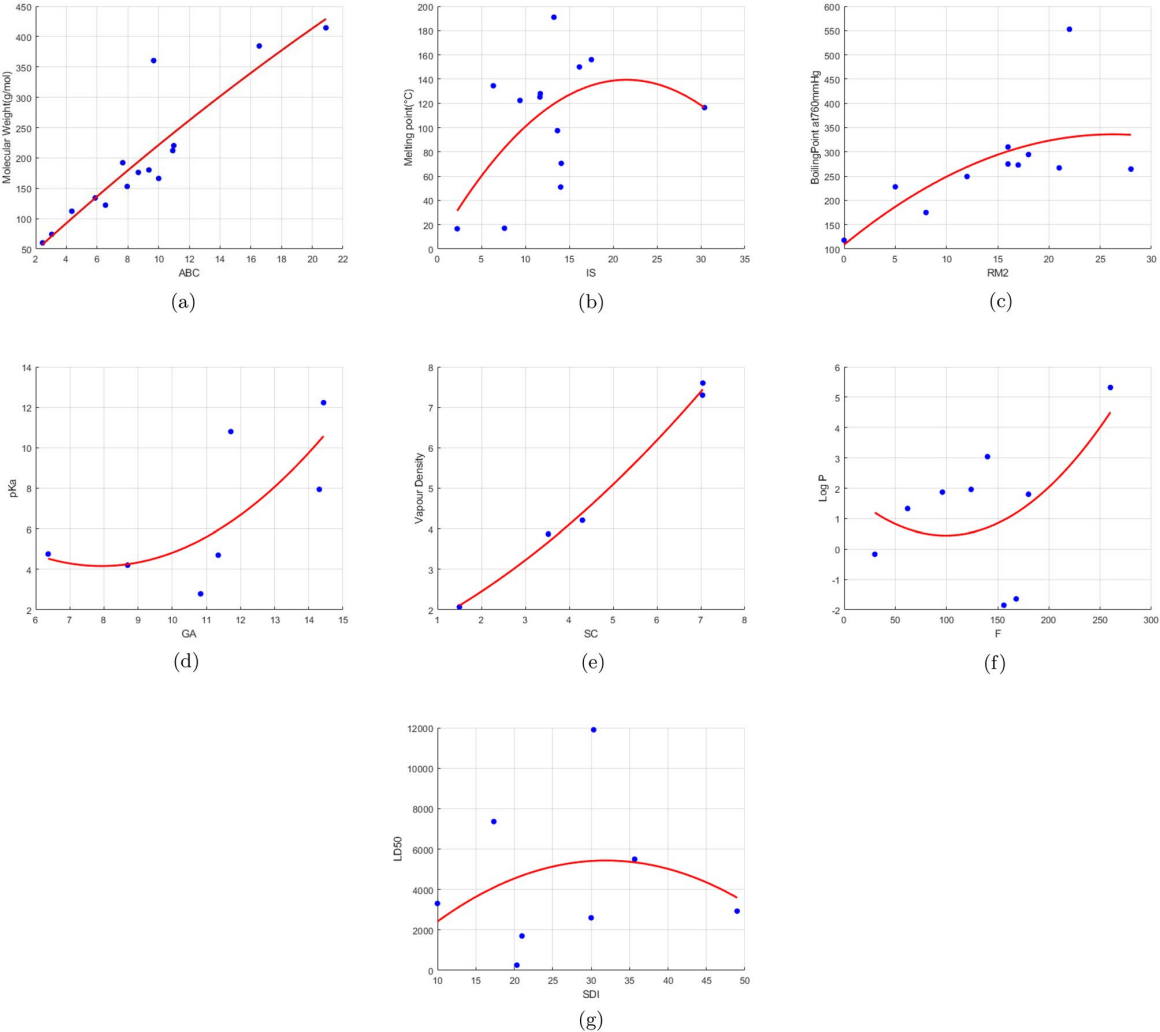
Quadratic regression plots.

In quadratic regression analysis, the Atom bond connectivity index has the highest predictive ability (highest regression coefficient) for molecular weight. Geometric arithmetic index and inverse sum connectivity index have the highest predictive ability for pKa value and melting point, respectively. Symmetric division index shows the highest predictive ability for LD50 value. Unlike linear regression, RM_2_ shows high predictive ability for boiling point. Forgotten index and sum connectivity index have the highest predictive ability for log P value and vapour density respectively. The regression coefficient between each property and index is displayed in Table 13. The quadratic regression plots of the physicochemical property and index pairs with the highest coefficient of determination are displayed in Fig. 5.

**Table 13:** Quadratic regression coefficients of properties with molecular descriptors.

	M${_1}$	M${_2}$	RM${_2}$	HM	AZ	R	RR	RRR	H	SC	GA	IS	F	SDI	ABC	So
MW	0.9022	0.8919	0.8607	0.8817	0.8899	0.8972	0.9024	0.8910	0.8907	.8980	0.8950	0.8904	0.8635	0.8952	**0.9038**	0.9003
MP	0.4622	0.4787	0.4913	0.4883	0.4693	0.4580	0.4653	0.4775	0.4683	0.4635	0.4677	**0.5403**	0.5069	0.4445	0.4533	0.4618
BP	0.6446	0.6551	**0.8630**	0.6616	0.6223	0.5949	0.6387	0.6277	0.5878	0.6028	0.6057	0.6340	0.6625	0.6440	0.6266	0.6483
pKa	0.7061	0.7104	0.6676	0.7223	0.5134	0.5418	0.6918	0.5752	0.4698	0.5733	**0.7258**	0.5632	0.7207	0.6839	0.6678	0.7084
VD	0.9899	0.9870	0.93840	0.9858	0.9847	0.9984	0.9918	0.9816	0.9893	**0.9974**	0.9945	0.9719	0.9867	0.9932	9949	0.9892
log P	0.5513	0.5323	0.4546	0.5480	0.4609	0.4660	0.5447	0.5030	0.4487	0.4979	0.5159	0.4339	**0.5528**	0.5340	0.5382	0.5504
LD50	0.2221	0.1222	0.2221	0.479	0.1600	0.192	0.2028	0.1545	0.1805	0.1907	0.1733	0.2338	0.2778	**0.2817**	0.2223	0.2364

Bold values indicate the highest correlation.

**Fig. 6:**
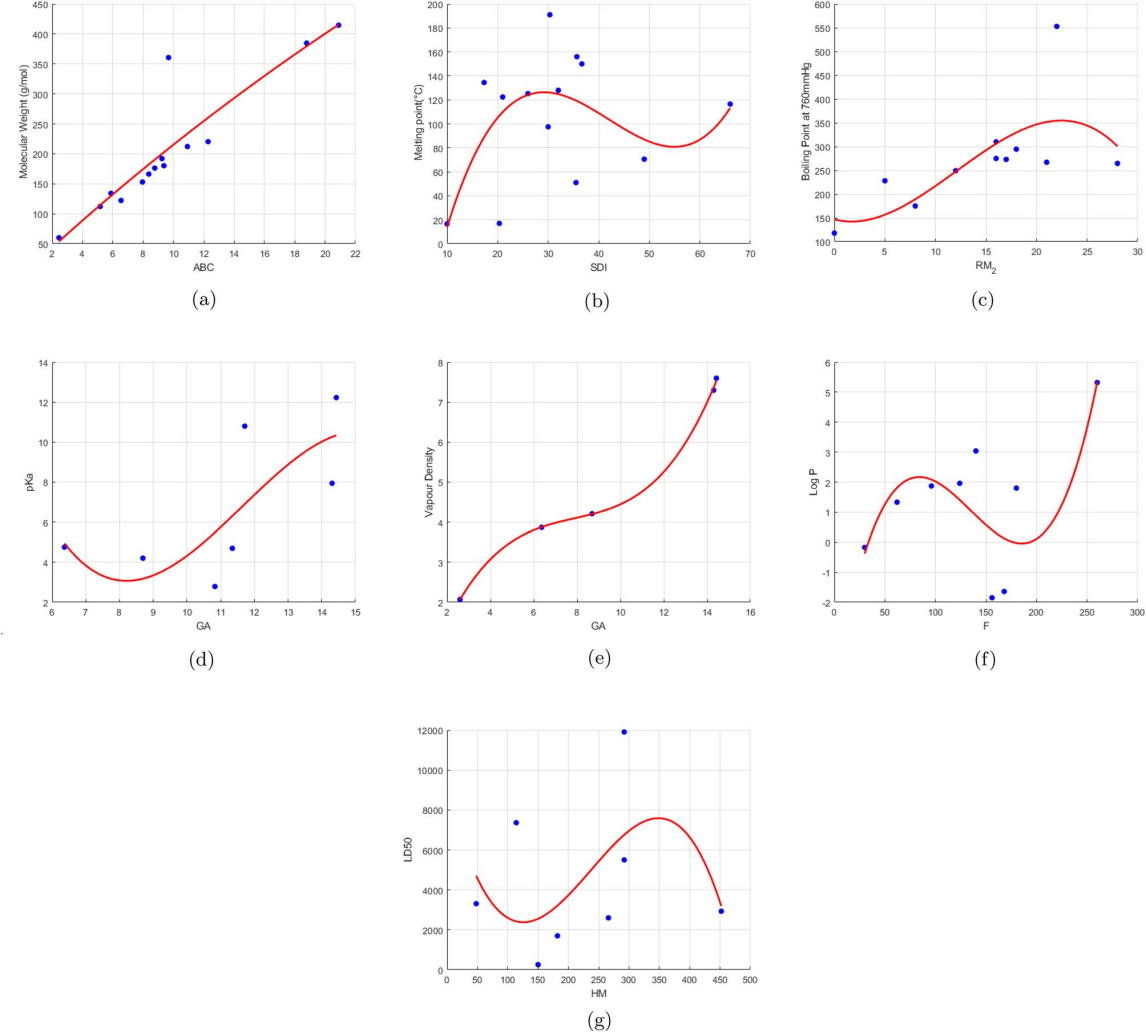
Cubic regression plots.

In cubic regression analysis, the Atom bond connectivity index exhibits the greatest predictive capacity, indicated by the highest regression coefficient, for molecular weight. The best predictor of pKa value and vapour density is the geometric arithmetic index. The best predictor of LD50 value and melting point is the symmetric division index. High boiling point prediction ability is demonstrated by RM_2_. The best predictor of the log P value is the forgotten index. The regression coefficient between each property and index is displayed in Table 14. The cubic regression plots of the physicochemical property and index pairs with the highest coefficient of determination are displayed in Fig. 6.

**Table 14:** Cubic regression coefficients of properties with molecular descriptors.

	M${_1}$	M${_2}$	RM${_2}$	HM	AZ	R	RR	RRR	H	SC	GA	IS	F	SDI	ABC	So
MW	0.9022	0.8920	0.8611	0.8818	0.8908	0.8972	0.9026	0.8911	0.8908	0.8981	0.8951	0.8904	0.8635	0.8987	**0.9039**	0.9004
**MP**	0.5681	0.5573	0.5286	0.5658	0.5189	0.5272	0.5608	0.5371	0.5202	0.5367	0.5351	0.5427	0.5669	**0.5744**	0.5634	0.5711
BP	0.6759	0.6949	**0.9071**	0.6797	0.6790	0.6779	0.6827	0.6645	0.6951	0.6826	0.6626	0.6531	0.6659	0.6455	0.6649	0.6698
pKa	0.7142	0.7160	0.6896	0.7224	0.5223	0.7051	0.7131	0.5765	0.4709	0.6711	**0.9419**	0.7171	0.7217	0.6987	0.7154	0.7125
VD	0.9918	0.9881	0.9842	0.9888	0.9848	0.9986	0.9920	0.9919	0.9940	0.9991	**0.9998**	0.9759	0.9897	0.9976	9958	0.9923
log P	0.7385	0.7380	0.6887	0.7519	0.4892	0.6634	0.7361	0.5676	0.5289	0.6750	0.6803	0.4970	**0.7677**	0.7426	0.7418	7393
LD50	0.4046	0.4538	0.4656	**0.4950**	0.3162	0.4017	0.3845	0.2781	0.4675	0.3950	0.3521	0.2548	0.5101	0.4125	0.3680	0.4187

Bold values indicate the highest correlation.

For molecular weight, the cubic model has the highest $\hbox {R}^2$ value, 0.9039, suggesting that it explains most variance, with ABC as the best predictor. For melting point, cubic model has the highest $\hbox {R}^2$-value (0.5744) but has a high *p* value of 0.2852, making the model statistically weak. For the boiling point in the cubic model, $R^2 = 0.9071$ and *p* value < 0.05, making it the best model, with the predictor index RM_2_. For the pKa value, $R^2 = 0.9419$ for cubic model, showing the best predictive ability. For vapor density, the cubic model is the best ($R^2 = 0.9998$) with the lowest RMSE. All the models are highly significant. For log *P* values and LD50 values, the models are statistically insignificant due to high *p* values ( > 0.05).

To develop the most predictive model, cubic regression offers the maximum accuracy for the four of molecular parameters, molecular weight (MW), boiling point (BP), pKa, and vapour density (VD), as indicated by the highest $R^2$ values. The models for BP, MW, pKa, and VD have *p* values less than 0.05 in terms of statistical significance, indicating that they are dependable in forecasting these properties. However, because of their weaker correlations and statistical insignificance, the models for Melting Point (MP), LogP, and LD50 show lower $\hbox {R}^2$ values and higher *p* values, making them less accurate predictors. Table 15 shows the best predictive model.

**Table 15:** Best predictive model.

Equation	R	$\hbox {R}^2$	*p* value
$MW = 0.0011\, ABC^3 - 0.1985\, ABC^2 + 23.7013\, ABC - 2.9687$	0.9039	0.8170	0.0005
$BP = -0.0477\, {RM_2}^3 + 1.7262\, {RM_2}^2 - 5.4672\, {RM_2} + 146.7865$	0.9071	0.8999	0.0005
$pKa = -0.0454GA^3 + 1.5893GA^2 + -16.9039GA + 47.5869$	0.9419	0.8854	0.0060
$VD = 0.0094\, {GA}^3 - 0.2216\, {GA}^2 + 1.8685\, {GA} - 1.4546$	0.9998	0.9996	0.0262

**Fig. 7:**
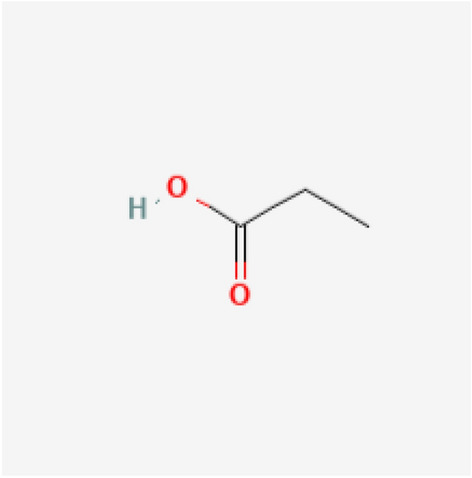
Propionic acid.

We take into consideration propionic acid, a different food preservative, in order to validate the predictive model. Propionic acid is a fungicide used to stop bacteria and fungi from growing in grain that has been stored for use by animals and poultry^58^. Propionic acid’s chemical structure is depicted in the Fig. 7. We compute the degree-based topological indices of propionic acid, as in Theorem 3.1, and the results are shown in Table 16. By substituting the index values in the predictive model, the physicochemical characteristics of propionic acid are estimated. The physiochemical characteristics’ experimental values are gathered from PubChem^45^. The experimental and predicted values for propionic acid’s physicochemical characteristics are compared in Table 17, which indicates that they are approximately equal. These results show how well the model predicts the characteristics of novel food preservative candidates. This technique can be used to screen a large number of chemicals and identify those that have a high potential for usage as food preservatives.

**Table 16:** Topological indices of Propionic acid.

M${_1}$	M${_2}$	RM${_2}$	HM	AZ	R	RR	RRR
16	14	2	66	22.75	2.2701	7.3278	1.4142
H	SC	GA	IS	F	SDI	ABC	So
2.0667	2.0246	3.6547	3.3667	38	11.3333	3.0472	12.1662

**Table 17:** Comparison between experimental and predicted values.

Property	Experimental value	Predicted value
Molecular Weight(g/mol)	74.08	78.2329
Boiling point (°C) at 760 mmHg	141	142.3753
pKa	4.88	4.82
Vapour Density	2.56	2.7514

## Conclusion

In this study, topological indices are effectively utilized to create prediction models for evaluating the physicochemical characteristics of food preservatives. We determined the most reliable method for predicting these attributes by comparing the linear and curvilinear regression models. From the findings, it can be concluded that cubic regression models are better than linear and quadratic models as they yield $R^2$ values such as 0.9998 for vapor density and 0.9039 for molecular weight, which indicates high predictive capability. The best predictive model is also identified. Furthermore, the applicability and dependability of the model are confirmed by its validation using propionic acid, an existing food preservative. In further studies, this predictive model can be utilized in the screening of food preservative compounds. This study demonstrates how computational approaches can be used to screen and design new food preservatives, providing an economical and effective substitute for experimental testing.

## Data Availability

All data generated or analysed during this study are included in this published article.
